# A Rare and Unexpected Side-Effect of Cannabis Use: Abdominal Pain due to Acute Pancreatitis

**DOI:** 10.1155/2015/463836

**Published:** 2015-02-11

**Authors:** Mehmet Husamettin Akkucuk, Mustafa Erbayrak

**Affiliations:** ^1^Department of Emergency Medicine, Başkent University School of Medicine, Saray Mahallesi Yunus Emre Caddesi No. 1, Alanya, 07400 Antalya, Turkey; ^2^Department of Gastroenterology, Başkent University School of Medicine, Saray Mahallesi Yunus Emre Caddesi No. 1, Alanya, 07400 Antalya, Turkey

## Abstract

Acute pancreatitis is a frequently encountered disorder in patients presenting to emergency units. Biliary system disorders, alcohol consumption, infections, and drugs are among the causes of acute pancreatitis. However, it is sometimes difficult to determine the etiology of this disorder, particularly if the patient does not wish to disclose his consumption of cannabis, the use of which is illegal.

## 1. Introduction

Acute pancreatitis is an inflammatory disorder of pancreas, caused mainly by biliary system disorders, alcohol consumption, and infections [1, 2]. Acute pancreatitis may also be caused by some drugs such as antibiotics (metronidazole), immunosuppressants (azathioprine), antihypertensives (angiotensin converting enzyme inhibitors, furosemide or thiazide diuretics), aspirin, or valproic acid [1, 3]. Case of acute pancreatitis due to cannabis has been rarely reported in the literature [4]. In this paper, because of its rarity, a case of cannabis-induced acute pancreatitis has been presented.

## 2. Case

A 44-year-old male patient presented to our Emergency Department with complaints of abdominal pain and nausea. He stated that he had been suffering from abdominal pain for one week. In other medical centers he had formerly visited, he had been told that his pain was due to a stomach disorder and, for therapy, a proton pump inhibitor had been commenced. Upon continuation of his complaints, the patient had presented to our Emergency Unit.

At the time of presentation, the patient had epigastric tenderness, but the other systemic findings were normal. His arterial blood pressure was 130/80 mm Hg, pulse was 86/minute, and body temperature was 37°C. His blood tests were as follows: WBC, 12.800/*µ*L, CRP, 0.60 mg/L, and serum electrolytes, serum calcium, AST, ALT, GGT, and bilirubin within their normal ranges. However, his serum amylase and lipase were 294 IU/L and 935 U/L, respectively (Ranson criteria score: 0). Dual testing with lipase and amylase had a sensitivity of 93% for pancreatitis [5]. The patient gave no history of a chronic disease, regularly medications used, alcohol consumption, or trauma. He had no history of a recent febrile disease, either. However, one of the family members stated that the patient was regularly taking cannabis powder that he had been preparing for a long time and stopped cannabis when his pains began.

On the abdominal computed tomography scan performed to determine the etiology and pancreas damage, there were no abnormalities in the pancreas, no peripancreatic fluid (Balthazar grade A acute pancreatitis), no gall stones, and no dilation in the biliary system. Following a consultation, the patient was hospitalized in the Clinic of Gastroenterology. Endoscopy revealed grade B esophagitis, two ulcers of 5 and 8 mm in the antrum, and a 1 cm ulcer in the duodenum. The computed tomography showed no penetration of the duodenal ulcer to the pancreas.

On the 3rd day of hospitalization, the patient's amylase and lipase levels were normalized, and, thereupon, the patient, who was thought to have had cannabis-induced pancreatitis, was discharged from the hospital with the necessary recommendations.

## 3. Discussion

Cannabis is a plant belonging to the Cannabaceae family. The active substances of cannabis are cannabinoids in resin, which are obtained through dried and then powdered leaves of the plant. The major cannabinoid in hashish is tetrahydrocannabinol (THC), which is responsible for the pharmacological effect of hashish. Powder hashish is obtained by drying the high THC-containing parts of female cannabis in the shade and then by grinding and sifting the dried parts.

The mechanism by which THC causes acute pancreatitis has not been fully clarified [4, 6]. There are two cannabinoid receptors in the human body, namely, CBI and CBII; CBI is found in the central nervous system, peripheral endothelial cells, and smooth muscle cells, whereas CBII is found in macrophages. These two receptors are also present in the pancreatic tissue [6]. The receptors affect the gastrointestinal system both positively and negatively [7, 8]. By decreasing gastric acid and intestinal secretions, they delay the gastric emptying [6, 9].

Dembiński et al. [7] have shown that, via CBI receptors, cannabinoids increase the blood supply and DNA synthesis in the gastric mucosa and inhibit the inflammatory mediator interleukin-1*β*. Studies on mice with cerulein-induced pancreatitis have shown that administration of anandamide, a cannabinoid receptor agonist, to mice increases the severity of pancreatitis, but the reason for this effect is not clearly explained [10, 11]. Some authors think that this increase in severity may be due to the effect of cannabinoids on the pancreatic canal and Oddi's sphincter [1, 4, 10]. The effect of cannabinoids on gastric secretions and emptying can be the cause of gastric and duodenal ulcers present in our patient.

In our case, Naranjo score was +1; the mean of this score is possible adverse drug reactions [2].

We achieved this score from the answers of the questions shown below.

1. Are there previous conclusive reports on this reaction? = yes (+1 points).
2. Did the adverse reaction improve when the drug was discontinued? = yes (+1 points).
3. Are there alternative causes that could have caused the reaction? = yes (−1 points).

The other questions answers were as follows:* do not know or not done* (0 points).

In conclusion, patients may not give a history of cannabinoid use which is illegal. In order to determine the etiology in patients with acute pancreatitis attacks, the use of hashish should also be questioned. Hashish consumers present to the emergency units with frequently repeated pancreatitis attacks [4].
